# Supraglottic jet oxygenation and ventilation decreased hypoxemia during gastrointestinal endoscopy under deep sedation at high altitudes: a randomized clinical trial

**DOI:** 10.1186/s12871-022-01902-3

**Published:** 2022-11-14

**Authors:** Bailin Jiang, Yi Li, Deji Ciren, Ouzhu Dawa, Yi Feng, Ciren Laba

**Affiliations:** 1 Department of Anesthesiology, Tibet Autonomous Region People’s Hospital, No.18 Linkuo North Road, Lhasa, Tibet, China; 2 Department of Anesthesiology, Peking University People’s Hospital, No.11 Xizhimen South Street, Xicheng District, Beijing, China

**Keywords:** Airway management, Gastrointestinal endoscopy, High altitudes, Hypoxia, Sedation, Supraglottic jet oxygenation and ventilation (SJOV), Wei nasal jet tube (WNJ)

## Abstract

**Background:**

Hypobaric hypoxia is common at high altitudes. Whether this exacerbates hypoxia during procedural sedation and whether hypoxia can be alleviated by the use of supraglottic jet oxygenation and ventilation (SJOV) are unknown. This study aimed to compare the incidence of hypoxia during gastrointestinal endoscopy under deep sedation at high altitudes with oxygen supply techniques using either a nasal cannula or SJOV.

**Methods:**

This study was conducted from April 2022 to July 2022 in a tertiary hospital located 3650 m above sea level. Adult patients scheduled for routine gastrointestinal endoscopy under sedation were enrolled and randomized 1:1 to receive SJOV or a nasal cannula during sedation. Moderate hypoxia was the primary outcome, defined as an SPO_2_ of 75–89% for < 60 s. The secondary outcomes were respiratory-, cardiovascular-, and SJOV-related complications. The influence of characteristics regarding acclimatization to high altitudes (Tibetan ethnic group and erythrocytosis) on the occurrence of hypoxia was analyzed.

**Results:**

None of the patients were lost to follow-up. A total of 72 patients were included in the analysis (36 patients in each group). There were 20 (27.8%) patients who experienced moderate hypoxia events. Significantly fewer hypoxic events occurred in the SJOV group than in the nasal cannula group [3 (8.3%) vs. 17 (47.2%), absolute risk difference (95% CI): − 38.9 (− 57.5, − 20.2) %, risk ratio (RR, 95% CI): 0.18 (0.06, 0.55), *P* < 0.001]. Significantly fewer patients in the SJOV group experienced mild hypoxia (P < 0.001) and severe hypoxia (*P* = 0.002). No serious adverse events occurred in either of the groups. The Tibetan ethnic group (*P* = 0.086) and erythrocytosis (*P* = 0.287) were not associated with the occurrence of hypoxia events.

**Conclusions:**

The incidence of hypoxia was lower with SJOV than with nasal cannula in patients undergoing gastrointestinal endoscopy under deep sedation at high altitudes. The Tibetan ethnic group and erythrocytosis did not influence the occurrence of hypoxia.

**Trial registration:**

This study was registered at ClinicalTrials.gov (NCT05304923) before enrollment by Dr. Yi Feng on 31/03/2022.

**Supplementary Information:**

The online version contains supplementary material available at 10.1186/s12871-022-01902-3.

## Introduction

Although a range of targeted levels of sedation may be available for gastrointestinal endoscopy [1], deep sedation is the consentaneous preference of patients and their endoscopists [2]. Deep sedation impairs the patient’s ventilatory function and may entail intervention to maintain a patent airway and adequate oxygenation/ventilation [1, 2], which may worsen at high altitudes. The drop in barometric pressure at high altitudes (≥2500 m) causes a conspicuous decrease in the partial pressure of oxygen, which leads to hypobaric hypoxia [3]. These defining environmental features pose substantial threats to hypoxemia in patients undergoing deep sedation at high altitudes. Supplemental oxygen administration via nasal cannula will not reduce the incidence of hypoxia in these patients, which might be adequate at low altitudes [1, 4].

Supraglottic jet oxygenation and ventilation (SJOV), a technique to maintain adequate oxygenation in patients with respiratory suppression or apnea [5], has been acknowledged as a potential approach to providing oxygenation/ventilation during unanticipated elective and/or emergency difficult airway management in the new American Society of Anesthesiologists Practice Guidelines [6]. SJOV can improve oxygenation with minimal barotrauma complications often seen during emergent transtracheal jet ventilation (TTJV) [6, 7]. It has been demonstrated that SJOV reduces the incidence of hypoxia during upper gastrointestinal endoscopy and improves patient safety [8]. Therefore, it is plausible that SJOV can reduce hypoxia during upper gastrointestinal endoscopy under deep sedation at high altitudes.

This study aimed to determine whether SOJV could reduce the incidence of hypoxia during gastrointestinal endoscopic procedures in deeply sedated patients at high altitudes compared to the commonly used supplemental oxygen administration via a nasal cannula. The influence of characteristics regarding acclimatization to high altitudes (Tibetan ethnic group and erythrocytosis) on the occurrence of hypoxia was also analyzed.

## Methods

### Study design

This was a single-center, parallel-group, prospective, double-blind, randomized clinical trial. The study was conducted from April 2022 to July 2022 at the Tibet Autonomous Region People’s Hospital, Lhasa, Tibet, China (3650 m above sea level). This hospital is a Class A tertiary comprehensive hospital and referral center in the Tibet Autonomous Region.

This study was registered at ClinicalTrials.gov (NCT05304923) before enrollment on 31/03/2022. Ethical approval for this study (approval number: ME-TBHP-22-02) was provided by the institutional review board of Tibet Autonomous Region People’s Hospital, Lhasa, Tibet, China (Chairperson Prof Ciren Dawa) on 21 April 2022. Written informed consent was obtained from all the participants. All personnel on the study team completed standardized training for procedural sedation and the use of SJOV. A separate investigator monitored the safety of the participants and recorded their outcomes. An independent monitoring group saved and analyzed the data as planned. This trial followed the Consolidated Standards of Reporting Trials (CONSORT) reporting guidelines.

### Patients

The patients were recruited from the Tibet Autonomous Region People’s Hospital. Patients could be eligible for the trial if they fulfilled the following inclusion criteria: a. 18 years or older; b. underwent routine gastrointestinal endoscopy under procedural sedation; c. consented to participate in this trial. The exclusion criteria included infection of the upper airway; anatomical abnormalities of the face, nose, and upper airway; coagulopathies; anticipated or known difficult airway; known allergy to propofol, soybeans, and egg; and absence from the high-altitude environment during the past 3 months. As per the instructions of the center, patients with severe disease will not be allowed to accept procedural sedation; thus, patients with severe pulmonary disease were also excluded from this study.

### Randomization and blinding

The participants were randomly allocated to either SJOV or nasal cannula oxygen supply in a 1:1 ratio using block randomization with variable block sizes of four or six randomized. Randomization was stratified according to whether upper endoscopy or colonoscopy (1:1) was planned. An independent research assistant generated randomization using a computer-generated list and assigned allocations. The group assignments were concealed from the investigators who enrolled the participants until after enrollment using sequentially numbered opaque envelopes. Participants and statisticians who assessed the outcomes were blinded. Since the clinicians and investigators had to conduct sedation and record the outcomes throughout the procedures, they could not be blinded.

### Intervention and procedure

In all patients, a loading dose of propofol (1 mg/kg) was administered, followed by an incremental bolus (10 mg) to titrate to a deep sedation level. To control for bias, no other sedation techniques were used in this study. Deep sedation was defined as a purposeful response after repeated or painful stimulation [1] and was approximately equivalent to the Observer’s Assessment of Alertness/Sedation (OAA/S) scale of 1 or 2 (1–5, 1 = deep sleep, 5 = alert) [9, 10]. Sedation was performed by the same anesthesiologist who had completed standardized training for procedural sedation and the use of SJOV. Another member of the study team supervised all the procedures. The OAA/S scale was administered every 2 minutes, and incremental boluses were titrated to maintain deep sedation (OAA/S scale< 3). Before sedation, each patient was oxygenated via a nasal cannula for two minutes to ensure that the peripheral oxygen saturation (SPO_2_) was ≥95%. No premedication was used in this study. After sedation, patients were positioned in the left lateral recumbent.

**Fig. 1:**
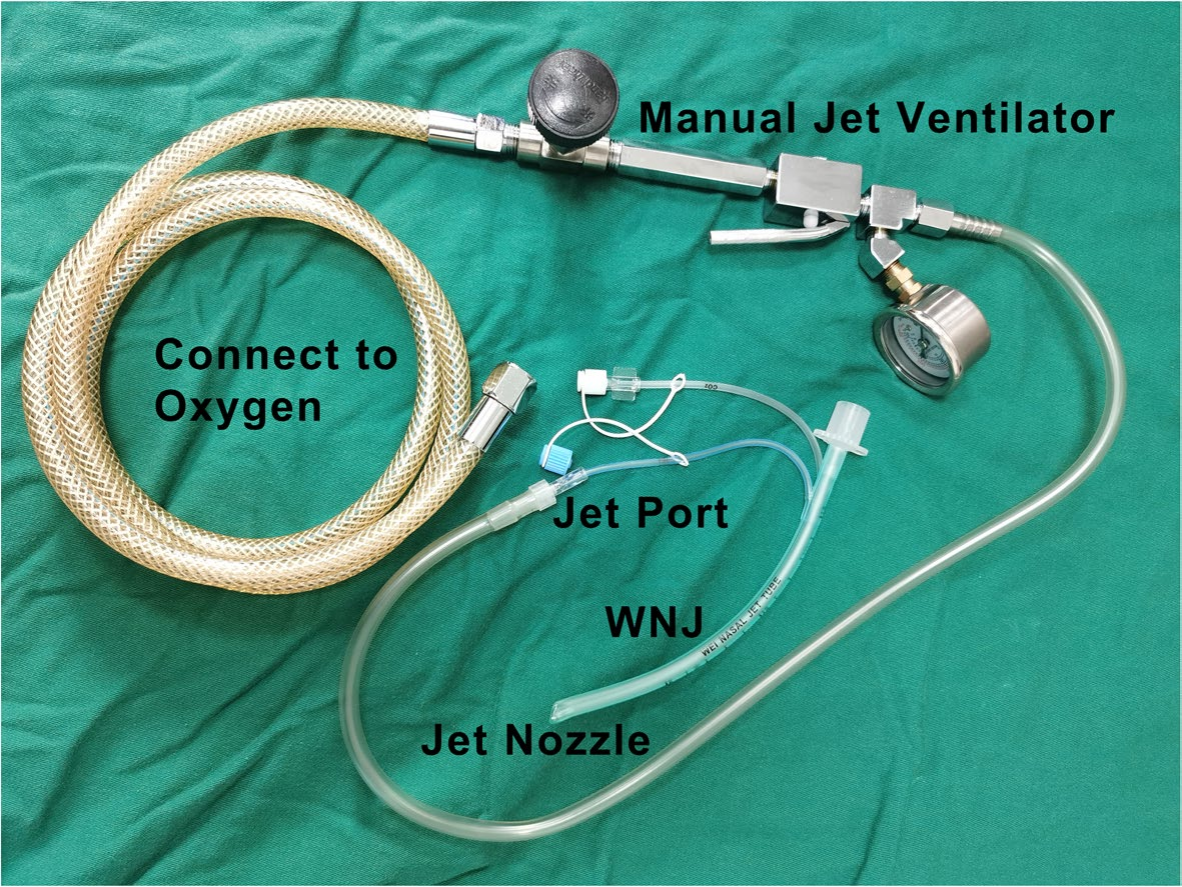
Supraglottic jet oxygenation and ventilation using a Wei nasal jet tube . SJOV was conducted using a WNJ connected to a manual jet ventilator via its jet port. The WNJ is a regular nasopharyngeal airway with two modified nozzles. One is the jet nozzle connected to the manual jet ventilator. Another nozzle is used to monitor breathing connected to a PaCO2 monitor (breathing was not monitored in this study). The regular nasal cannula could also supply up to 6 l/minute of oxygen. Only the jet nozzle was used for oxygen administration in this study. SJOV, supraglottic jet oxygenation and ventilation; WNJ, Wei nasal jet tube

In the nasal cannula oxygen supply group, oxygen supplementation at 2 l/min was delivered via a nasal cannula. In the SJOV group, SJOV was conducted using a Wei nasal jet tube (WNJ, Well Lead Medical Co. Ltd., Guangzhou, China), which was connected to a manual jet ventilator (Well Lead Medical Co. Ltd., Guangzhou, China) via its jet port (Fig. 1) [8, 11]. The initial settings of SJOV were as follows: driving pressure (DP) 15 psi; respiratory rate (RR) 20 bpm; inspiratory-to-expiratory (I/E) ratio 1:2, and gas supply, 100% oxygen [8]. The WNJ was inserted through the nostril toward the vocal cords, and chest excursion was visible when the ideal placement was achieved, albeit not necessarily [8].

After the target sedation level was reached, the WNJ was placed in lieu of the nasal cannula to initiate SJVO in the SJOV group. Before the patients recovered from sedation, the WNJ was replaced with a nasal cannula to ensure that the patients were blinded to the assignment.

When SPO_2_ was < 95%, increasing oxygen supplementation was performed first. In the nasal cannula oxygen supply group, the oxygen flow rate was increased to 6 l/min. In the SJOV group, the RR was adjusted to 30 bpm, and the DP was increased to 20 psi. If SPO_2_ fell below 90%, the airway was opened with a jaw-thrust maneuver in both groups [12]. If hypoxemia could not be corrected within 1 min or SPO_2_ was < 75%, mask ventilation was considered in both groups [8, 9]. Tracheal intubation can be performed, if necessary. No other additional airway devices were used in this study.

Blood pressure, heart rate, and pulse oximetry were recorded before oxygen supplementation as the baseline and repeated every 2 min. Hemodynamic management and administration of additional analgesics (alfentanil in this study) were performed at the discretion of the anesthesiologists based on the patients’ clinical signs. The total propofol dose, total alfentanil dose, procedure duration, and hypoxia-related interventions were recorded. Patients in both groups were monitored for 20 min after awakening.

### Outcomes

The primary outcome was moderate hypoxia during sedation, defined as an SPO_2_ of 75–89% for < 60 s [8]. The secondary outcomes were adverse events except for moderate hypoxia, which included respiratory-related complications including pulmonary aspiration, mild hypoxia (SPO_2_ = 90–95%) [8], and severe hypoxia (SPO_2_ < 75% or < 90% for > 60 s) [8]; cardiovascular-related complications including hypotension (systolic blood pressure < 90 mmHg), hypertension (systolic blood pressure > 160 mmHg), bradycardia (heart rate < 50 beats/min), tachycardia (heart rate > 120 beats/min); and fatal complications including severe anaphylactic reactions, myocardial infarction, cardiac arrest, and death [8, 13, 14]. Adverse events related to SJOV were also recorded 20 min after the patient awoke, which comprised pharyngalgia, xerostomia, nasal bleeding, and barotrauma.

### Sample size calculation

The sample size was estimated based on the primary outcome of hypoxia. There have been no reports of hypoxemia during deep sedation during gastrointestinal endoscopy at high altitudes. In a study on hypoxemia during moderate sedation for gastrointestinal endoscopy at low altitudes [8], the incidences of hypoxia were 9% when a nasal cannula was used to supply oxygen and 3% when SJOV was used. The incidence of sedation-related hypoxia in gastrointestinal endoscopy varies considerably [8, 12, 14, 15]. Given the deeper sedation level and higher altitude, we assumed that 30% of patients in the nasal cannula oxygen supply group might encounter hypoxia, which was still within the reported range [12, 14] and consistent with our unpublished preliminary data. Considering the ventilation competence of SJOV [5, 11, 16], the incidence of hypoxia might not deviate from the reported rate (3%). We assumed an incidence of 5% in the SJOV group, which corresponded to an absolute difference of 25% between the groups. With these estimates, a 2-sided α level of 5%, and an attrition rate of 10%, a total of 36 patients per group (72 in total) were required to have 80% power to show a significant difference between groups.

### Statistical analysis

The patients were analyzed according to the randomization group. Multiple imputations were planned for missing data. Continuous variables were expressed as the median with interquartile range (IQR) or mean with standard deviation (SD), as appropriate. Categorical data are presented as numbers and proportions and were compared using the chi-square test or Fisher’s exact test, as appropriate. A 2-sided *P* value of less than 0.05 was considered statistically significant. A modified Poisson regression model was used to adjust the primary outcome for the stratification variable (upper endoscopy or colonoscopy) and baseline characteristics [age, sex, ethnic group, BMI (body mass index), history of OSAHS (obstructive sleep apnea-hypopnea syndrome), erythrocytosis, original hypoxia before oxygenation, procedure time, total propofol dose, and total alfentanil dose]. Subgroup analyses were not conducted because of the limited sample size. Sensitivity analyses were established a priori and performed using alternate definitions of the primary outcome. The alternate definitions (Supplementary Table 1) comprised mild to moderate hypoxia (SPO_2_ of 90–95% or 75–89% for < 60 s), moderate to severe hypoxia (SPO_2_ < 89%), and mild to severe hypoxia (SPO_2_ < 95%). Because of the potential for type I errors due to multiple comparisons, findings for analyses of secondary outcomes should be interpreted as exploratory. All statistical analyses were performed using IBM SPSS Statistics (version 25.0; IBM Corp., Armonk, NY, USA).

## Results

### Patients

**Fig. 2:**
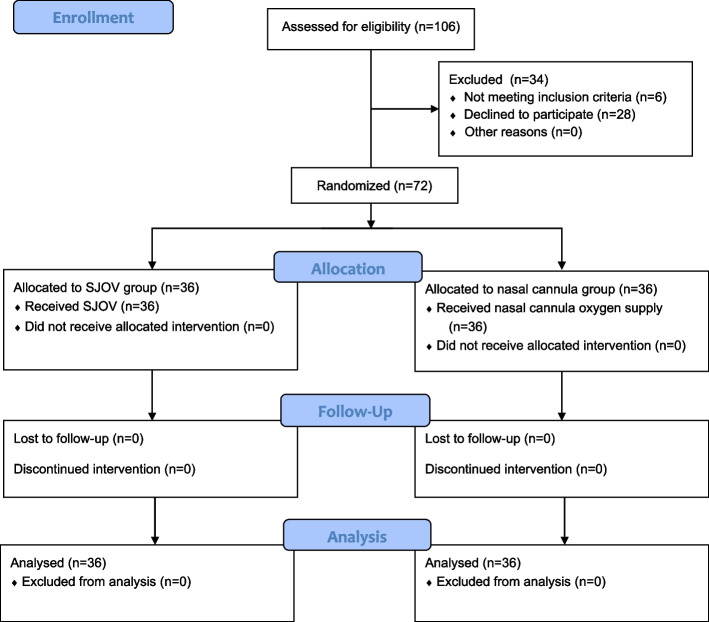
Flow diagram of patients included in this study

A total of 106 patients were screened for eligibility, of whom 72 were randomized. None of the participants were lost to follow-up. All participants [median (IQR) age, 49.0 (43.0, 56.8) years; 37 (51.4%) male; 45 (62.5%) Tibetan] were included in the full analysis set, with 36 participants (50%) randomly assigned to the SJOV group and 36 participants (50%) to the nasal cannula group (Fig. 2). No data were missing. Baseline characteristics were similar across the groups (Table 1). Erythrocytosis, defined as a hemoglobin concentration of > 165 g/L in men and > 160 g/L in women, was common among the participants [32 participants (44.4%)]. Original hypoxia, defined as peripheral oxygen saturation < 90% before oxygenation, was also common among participants [25 participants (34.7%)]. All patients were successfully oxygenated (SPO2 ≥ 95%) before sedation via a nasal cannula at 2 l/min. No hypoxia occurred during the recovery phase.

**Table 1:** Baseline characteristics of patients

Characteristic	SJOV (*n* = 36)	Nasal cannula (n = 36)
Age (year)	48.5 (42.0–57.0)	50.0 (44.8–55.8)
BMI (kg/m^2^)	23.7 ± 2.6	22.8 ± 2.8
Male [*n* (%)]	18 (50.0)	19 (52.8)
Tibetan [*n* (%)]	24 (66.7)	21 (58.3)
ASA grade of III or severer [*n* (%)]	3 (8.3)	4 (11.1)
Comorbidity [*n* (%)]		
Hypertension	6 (16.7)	12 (33.3)
Coronary artery disease	4 (11.4)	1 (2.8)
Diabetes	0 (0.0)	1 (2.8)
COPD	2 (5.6)	0 (0.0)
Asthma	2 (5.6)	2 (5.6)
OSAHS	6 (16.7)	6 (16.7)
Total	14 (38.9)	17 (47.2)
Hemoglobin concentration (g/L)	161.5 ± 22.5	157.8 ± 29.0
Erythrocytosis [*n* (%)] ^a^	15 (41.7)	17 (47.2)
Baseline vital signs		
Peripheral oxygen saturation (%)	90.3 (2.2)	90.3 (2.8)
Original hypoxia [*n* (%)] ^b^	12 (33.3)	13 (36.1)
Mean blood pressure (mmHg)	88.7 ± 18.0	96.1 ± 19.2
Heart rate (beat/mi)	71.3 ± 13.2	73.0 ± 13.0
Procedural characteristics		
Procedure time (min)	11.0 (8.5–14.8)	10.5 (8.0–15.0)
Total propofol dose (mg)	120 (70–150)	115 (85–150)
Total alfentanil dose (ug)	500 (0–1000)	500 (0–725)

Continuous variables are expressed as mean ± SD or median (IQR), as appropriate; categorical data are presented as the number of events (proportion)

*SJOV* supraglottic jet oxygenation and ventilation, *BMI* body mass index, *ASA* American Society of Anesthesiology classification of physical status, *COPD* chronic obstructive pulmonary disease, *OSAHS* obstructive sleep apnea-hypopnea syndrome

^a^Erythrocytosis was defined as a hemoglobin concentration of > 165 g/L in men and > 160 g/L in women; ^b^ Original hypoxia was defined as peripheral oxygen saturation of < 90% before oxygenation

### Primary outcome

Twenty (27.8%) participants experienced moderate hypoxia events, 14 (38.9%) underwent upper endoscopy, and 6 (16.7%) underwent colonoscopy. Significantly fewer hypoxic events occurred in the SJOV group than in the nasal cannula group (table 2).

**Table 2:** Primary and Secondary Outcomes

Outcome	SJOV (*n* = 36)	Nasal cannula (*n* = 36)	Absolute risk difference, (95% CI), %	SJOV vs. nasal cannula, Risk ratio (95% CI)	*P* value
**Primary outcome**					
Moderate hypoxia [*n* (%)]	3 (8.3)	17 (47.2)	−38.9 (−57.5, − 20.2)	0.18 (0.06, 0.55)	< 0.001
**Secondary outcomes**					
Respiratory-related [*n* (%)]					
Mild hypoxia	5 (13.9)	23 (63.9)	−50.0 (− 69.3, −30.7)	0.22 (0.09, 0.51)	< 0.001
Severe hypoxia	0 (0.0)	9 (25.0)	−25.0 (−39.1, −10.9)	–	0.002
Pulmonary aspiration	0 (0.0)	0 (0.0)	–	–	–
Rescue events [*n* (%)]					
Jaw-thrust	3 (8.3)	16 (44.4)	−36.1 (−54.7, −17.5)	0.19 (0.06, 0.59)	0.001
Mask ventilation	0 (0.0)	6 (16.7)	−16.7 (−28.8, −4.5)	–	0.025
Tracheal intubation	0 (0.0)	0 (0.0)	–	–	–
Cardiovascular-related [*n* (%)]					
Hypotension	7 (19.4)	7 (19.4)	0 (−18.3, 18.3)	1.00 (0.39, 2.56)	1.000
Outcome	SJOV (*n* = 36)	nasal cannula (n = 36)	Absolute risk difference, (95% CI), %	SJOV vs. nasal cannula, Risk ratio (95% CI)	*P* value
Hypertension	3 (8.3)	1 (2.8)	5.6 (−4.9, 16.1)	0.33 (0.04, 3.06)	0.614
Bradycardia	8 (22.2)	11 (30.6)	−8.3 (−28.6, 11.9)	0.73 (0.33, 1.59)	0.422
Tachycardia	0 (0.0)	0 (0.0)	–	–	–
Fatal complications [*n* (%)] ^a^	0 (0.0)	0 (0.0)	–	–	–
SJOV-related [*n* (%)] ^b^					
Pharyngalgia	1 (2.8)	0 (0.0)	2.8 (−2.6, 8.1)	–	1.000
Xerostomia	1 (2.8)	0 (0.0)	2.8 (−2.6, 8.1)	–	1.000
Nasal bleeding	3 (8.3)	0 (0.0)	8.3 (−0.7, 17.4)	–	1.000
Barotrauma	0 (0.0)	0 (0.0)	–	–	–

*SJOV* supraglottic jet oxygenation and ventilation, *CI* confidence interval

^a^Fatal complications embracing severe anaphylactic reactions, myocardial infarction, cardiac arrest, and death; ^b^Adverse events related to SJOV were also recorded 20 min after patients awoke

**Fig. 3:**
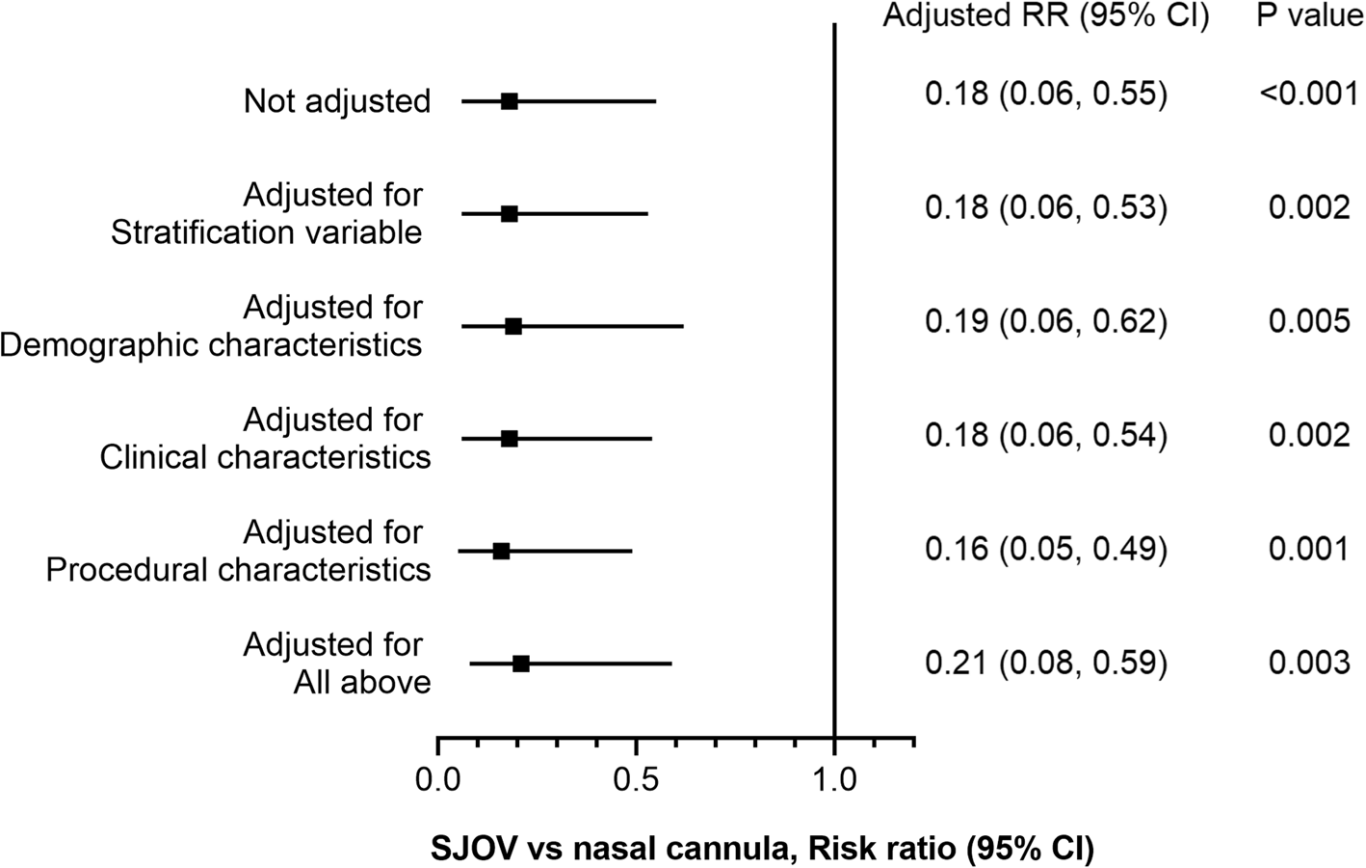
The primary outcome was adjusted for the baseline characteristics. Stratification variable: Upper endoscopy or colonoscopy; demographic characteristics: age, sex, ethnic group (Tibetan/Zang or not), body mass index, clinical characteristics, history of obstructive sleep apnea-hypopnea syndrome, erythrocytosis, and original hypoxia before oxygenation. RR, risk ratio; CI, confidence interval; SJOV, supraglottic jet oxygenation and ventilation

Following adjustments for stratification variables (upper endoscopy or colonoscopy), demographic characteristics (age, sex, ethnic group [Tibetan/Zang or not], BMI), clinical characteristics (history of OSAHS, erythrocytosis, original hypoxia before oxygenation), and procedural characteristics (procedure time, total propofol dose, total alfentanil dose), significantly lower incidences of moderate hypoxia were consistently associated with the use of SJOV, with a negligible fluctuation in the RRs (Fig. 3).

### Secondary outcomes

The secondary outcomes are shown in Table 2. There were significantly fewer participants who experienced mild hypoxia and severe hypoxia in the SJOV group than in the nasal cannula group. Consequently, significantly fewer rescue events were performed in the SJOV group, including jaw-thrust and mask ventilation.

There was no significant difference between the groups in terms of cardiovascular and SJOV-related complications. No serious adverse events occurred in either group, including pulmonary aspiration, barotrauma, severe anaphylactic reactions, myocardial infarction, cardiac arrest, or death (Table 2).

### Ancillary analyses

Alternate definitions of the primary outcome did not alter the inferences of this study. The use of SJOV was consistently protective against hypoxia (Supplementary Table 1).

A post hoc analysis using a modified Poisson regression model to adjust the moderate hypoxia for procedural characteristics revealed that the total propofol dose was significantly associated with the occurrence of moderate hypoxia (*P* = 0.047), but the total alfentanil dose was not associated with the occurrence of moderate hypoxia (*P* = 0.416). The modified Poisson regression model adjusting the moderate hypoxia for demographic and clinical characteristics revealed that the Tibetan ethnic group (*P* = 0.086), erythrocytosis (*P* = 0.287), and original hypoxia before oxygenation (*P* = 0.429) were not associated with the occurrence of moderate hypoxia events.

## Discussion

This study reports the incidence of hypoxic events during deep sedation for gastrointestinal endoscopy at high altitudes and confirms the merit of SJOV in ameliorating hypoxia. Given its minor clinical significance, mild hypoxia (SPO_2_ = 90–95%) [8] was exploratory in this study. Moderate hypoxia (SPO_2_ of 75–89% for < 60 s) [8] was used as the primary outcome, and moderate to severe hypoxia (SPO_2_ < 89%) was used as an alternative definition of the primary outcome for sensitivity analysis. The study was conducted in Lhasa, which is 3650 m above sea level. To the best of our knowledge, no relevant data have been reported for such high altitudes. High altitudes have resulted in several physiological and pathological changes in the inhabitants, [3, 17–19] which complicates hypoxia. To reduce the influence of potential confounders, patients who had been locally inhabited for no more than three months were excluded. Intriguingly, the Tibetan ethnic group, erythrocytosis, and original hypoxia before oxygenation, which might intuitively influence the hypoxia attributable to acclimatization, [20] did not alter the occurrence of hypoxia or the boon of SJVO against hypoxia.

Although the ramifications of high-altitude hypoxia might have been well appreciated [3, 19–23], there have been few reports on the influence of high-altitude hypoxia on anesthesia management. Compared with previous studies, [8, 24–27] the occurrence of moderate hypoxia was more common in the nasal cannula group (47.2%). Strategy behooves to reduce such a high incidence, which poses a substantial threat to patients. SJOV is an oxygen therapy for the treatment of hypoxia [5] and has been demonstrated as an effective approach to tackle hypoxia during moderate-deep sedation for gastroscopy at low altitudes [8]. In this study, SJOV reduced the incidence of moderate hypoxia from 47.2 to 8.3%, albeit higher than at low altitudes (3%) [8]. The high altitudes weakened the role of SJOV in preventing hypoxemia. However, given the high morbidity of hypoxia in the control group and before oxygenation, the decrease in the incidence of hypoxia supported the role of SJOV. Moreover, SJOV exempted patients from severe hypoxia, which occurred in 25% of patients in the nasal cannula group; consequently, no rescue manipulation that interrupted upper endoscopy was performed in the SJOV group. Therefore, SJOV was demonstrated to be an effective approach for treating hypoxia at high altitudes and may be generalized to other outpatient procedures in high-altitude areas.

This study also focused on the influence of special characteristics regarding acclimatization to high altitudes on the occurrence of hypoxia. Whether these characteristics benefit the inhabitants by directly preventing hypoxia or some covert approaches for enhancing the tolerance to hypoxia is unknown. Two overriding characteristics regarding acclimatization were underlined in this study, including erythrocytosis and the Tibetan ethnic group. Erythrocytosis is considered a common aspect of acclimatization to high altitudes [3, 17, 18]. Due to some gene variants, Tibetan highlanders were believed to be superior to lowlanders regarding acclimatization to high-altitude hypoxia [20]. However, none of these variables were demonstrated to be associated with hypoxia in this study. Perhaps these might benefit patients by enhancing their tolerance to hypoxia. However, before this role is precisely addressed, a positive strategy against hypoxia is warranted.

Fewer adverse events related to SJOV, including pharyngalgia, xerostomia, and nasal bleeding, were observed compared to those in a previous study [8]. This might be due to the special preparation for WNJ in this study. The WNJ was soaked in lidocaine hydrochloride mucilage prior to insertion. This might alleviate tissue injury by lubricating the WNJ and providing local anesthesia that contributes to analgesia. Furthermore, the residents in this area had already acclimatized to a dry climate, which might at least partly mitigate the complaints. It should also be noted that although the patients were fully awake, the follow-up period (20 min after awakening) might not be long enough to detect SJOV-related adverse events. Since the topical anesthesia did not expire, the discomfort could be concealed. Studies with longer follow-up will address this issue in the future.

Moreover, an original mild hypoxia or even moderate hypoxia, according to the definition of this study, before oxygen supplementation was observed in the majority of patients (hypoxia was observed in approximately 35% of the patients before oxygen supplementation). This finding suggests that the clinical significance of hypoxia should be addressed. Acclimatization of hypoxia could possibly mitigate the detrimental effects on these patients but deviates from the aim of this study. Future studies should focus on these aspects.

Since no recommended definition of hypoxia at high altitudes has been attained, the definition of hypoxia at low altitudes was used in this study [8]. Both severe hypoxia (SPO_2_ < 75% or < 90% for > 60 s) and the alternate definitions of hypoxia (Supplementary Table 1) were compared between the groups. Similar results ensued. These analyses may free the concern about a too narrow and strict criterion of hypoxia at high altitudes. Furthermore, whether acclimatization to hypoxia can free patients from the detriment of mild hypoxia is unknown. This study indicated the need to explore the definition of hypoxia at high altitudes. Further studies are warranted to provide evidence for revising the definition of hypoxia at high altitudes.

When SPO_2_ was < 95%, the oxygen flow rate was increased to 6 l/min in the nasal cannula group. Hypoxia was compared between SJOV with a fraction of inspired oxygen (FiO_2_) of 100% and nasal cannula at 6 l/min (FiO_2_ approximately 44%). Future studies should consider SJOV with a lower FiO_2_ or compare SJOV with more powerful oxygenation methods, such as high-flow nasal oxygen therapy.

Furthermore, the duration of hypoxemia was not recorded in this study, which might have precluded detailing the impact of the event. However, owing to patient protection, rescue strategies against hypoxemia have been implemented. This confounded the natural process of hypoxemia, especially when a prudent anesthesiologist was engaged, and consequently weakened the importance of recording duration.

To mimic the real world, propofol and alfentanil were administered at the discretion of the anesthesiologists. However, the doses of propofol and alfentanil were similar across the groups. Although the total dose of propofol was associated with hypoxia, following adjustments for procedural characteristics, including the doses of propofol and alfentanil, the incidence of hypoxia was still lower in the SJOV group. This excluded the concern that the potential difference in propofol or alfentanil between the groups may alter the inference. The dose of alfentanil was not associated with hypoxia in this study, which may be due to its relatively low dose.

### Limitations

First, this was a single-center study, and intrinsic bias should be considered when interpreting the results. Second, owing to the limited sample size, no subgroup analysis was conducted. This might omit some information, especially regarding the stratification variable, since more hypoxia was observed during the upper endoscopy. However, modified Poisson regression analysis demonstrated that the stratification variable did not alter the inferences of this study.

## Conclusions

The incidence of moderate hypoxia during gastrointestinal endoscopy under deep sedation at high altitudes is conspicuously high and warrants further intervention. The incidence of hypoxia is lower in SJOV than in nasal cannulas in this population.

## Supplementary Information

**Additional file 1: Supplementary Table 1.** Adjusted and sensitivity analyses.

## Data Availability

The datasets generated and analyzed during the current study are available in the Figshare repository (10.6084/m9.figshare.20222085.v2).

## Abbreviations

SJOV: Supraglottic jet oxygenation and ventilation
TTJV: Transtracheal jet ventilation
CONSORT: Consolidated Standards of Reporting Trials
OAA/S: Observer’s Assessment of Alertness/Sedation
WNJ: Wei nasal jet tube
DP: Driving pressure
RR: Respiratory rate
I/E: Inspiratory-to-expiratory
SPO_2_: Peripheral oxygen saturation
IQR: Interquartile range
BMI: Body mass index
OSAHS: Obstructive sleep apnea-hypopnea syndrome
CI: Confidence interval
FiO_2_: Fraction of inspired oxygen
