# Supplementary material for: Supraglottic jet oxygenation and ventilation decreased hypoxemia during gastrointestinal endoscopy under deep sedation at high altitudes: a randomized clinical trial
Source: BMC Anesthesiol. 2022 Nov 14;22:348. doi: 10.1186/s12871-022-01902-3 (PMC9661813; doi:10.1186/s12871-022-01902-3)
Supplement: Supplementary file 1 — Additional file 1: Supplementary Table 1. Adjusted and sensitivity analyses. [file 12871_2022_1902_MOESM1_ESM.docx]

**Supplementary Table 1.** Adjusted and sensitivity analyses.

| Primary outcome | SJOV vs. nasal cannula, Risk ratio (95% CI) | *P* value |
| --- | --- | --- |
| Moderate Hypoxia ^a^ | 0.18 (0.06, 0.55) | <0.001 |
| Alternate definitions of primary outcome |  |  |
| Mild to moderate hypoxia ^b^ | 0.22 (0.09, 0.51) | <0.001 |
| Moderate to severe hypoxia ^c^ | 0.18 (0.06, 0.55) | <0.001 |
| Mild to severe hypoxia ^d^ | 0.22 (0.09, 0.51) | <0.001 |

SJOV, supraglottic jet oxygenation and ventilation;

^a^ SPO_2_ of 75–89% for< 60 s; ^b^ SPO2 of 90–95% or 75–89% for< 60 s; ^c^ SPO2 < 89%; ^d^ SPO2 < 95%.
